# The disturbance of thyroid-associated hormone and its receptors in brain and blood circulation existed in the early stage of mouse model of Alzheimer’s disease

**DOI:** 10.18632/aging.204570

**Published:** 2023-03-07

**Authors:** Bingxiu Ren, Jinxin Ma, Min Tao, Gongwei Jing, Sheng Han, Chengyi Zhou, Xin Wang, Jiaoya Wang

**Affiliations:** 1 Department of Nuclear Medicine, The Third Affiliated Hospital of Zunyi Medical University (The First People’s Hospital of Zunyi), Zunyi, Guizhou 563000, China; 2 Department of Hospital Infection Management, The Third Affiliated Hospital of Zunyi Medical University (The First People’s Hospital of Zunyi), Zunyi, Guizhou 563000, China

**Keywords:** thyroid hormone, Alzheimer's disease, animal model, okadaic acid, hippocampus

## Abstract

Background: Studies showed that thyroid function plays an important role in the pathology of Alzheimer’s disease (AD). However, changes in brain thyroid hormone and related receptors in the early stage of AD were rarely reported. The aim of this study was to explore the relationship between the early stage of AD and local thyroid hormone and its receptors in the brain.

Methods: The animal model was established by stereotactic injection of okadaic acid (OA) into hippocampal region for the experiment, and 0.9% NS for the control. Blood sample from each mouse was collected and then the mice were sacrificed and the brain tissue was collected for detecting free triiodothyronine (FT3), free thyroid hormone (FT4), and thyroid-stimulating hormone (TSH), thyrotropin-releasing hormone (TRH) and phosphorylated tau, amyloid-β (Aβ) and thyroid hormone receptors (THRs) in the hippocampus of the mice were detected as well.

Results: Enzyme-linked immunosorbent assay showed that compared with the control, FT3, FT4, TSH and TRH in brain were significantly increased in the experimental group; in the serum, FT4, TSH and TRH were increased, while FT3 had no change; western blot analysis indicated that the expression of THR α and β in the hippocampus of the experimental group was significantly higher than that of the control.

Conclusion: Based on the results of this study, a mouse AD model can be established successfully by injecting a small dose of OA into the hippocampus. We speculate that early AD brain and circulating thyroid dysfunction may be an early local and systemic stress repair response.

## INTRODUCTION

Alzheimer’s disease (AD) is a degenerative disease of the nervous system characterized by progressive memory loss and cognitive impairment. Aβ and P-tau are the core pathological substances of AD [1]. Thyroid hormone including 3,3′,5-triiodothyronine (T3) and thyroxine (T4), whose precursors are less active are essential hormones that promote normal nerve development and play an important role throughout life. Early thyroid hormone deficiency disease, also known as congenital hypothyroidism, is usually caused by iodine deficiency and a mother’s hypothyroidism. If left untreated after birth, it can have irreversible and serious consequences for cognitive function. In contrast, hypothyroidism in adults alters memory and motor coordination, increasing the risk of depression, but these symptoms can be reversed with alternative therapies [2]. Studies in rat and mouse models also showed that the absence of T3, which regulates target gene transcription via the thyroid hormone receptor THRs (THRα and THRβ) in the brain leads to developmental and physiological dysfunction in multiple brain regions [3–5]. Studies confirmed that thyroid hormone is closely related to the development of the nervous system and to the function of learning and memory; a decrease in thyroid hormone impairs the function of learning and memory. These lesions were characterized by changes in the induction and maintenance of long-term potentiation (LTP) and long-term inhibition (LTD) in the hippocampal CA1 region [6, 7].

There were many reports about the relationship between thyroid hormone and AD, and two different points of view exist in these reports. One is that thyroid dysfunction causes AD, and their main evidence is that diagnosed patients with Alzheimer disease were associated with thyroid hormone dysregulation and disturbed circadian rhythms [8, 9]; In addition, AD patients also accompanied by depression, irritability and other behavioral changes [10], cognitive impairment [11, 12], regional cerebral blood flow [12], brain glucose consumption reduction and so on were associated with thyroid hormone deficiency [13, 14]. The alternative view is that the development of AD causes changes in thyroid hormone levels, meaning that thyroid dysfunction may be a result of the disease, support for this hypothesis can be explained by secondary deficits in AD neurodegenerative features, such as a worsening of the pituitary gland, which can lead to a reduction in TRH and TSH production, the resulting low TH levels [15] are explained by a lack of response to normal physiological stimuli, including melatonin from the pineal gland, TRH, and melatonin from T4 [16, 17], based on hypophyseal degeneration. But there were few reports of animal studies or clinical studies that supported this view. Although previous studies have confirmed a link between thyroid hormone and AD, whether thyroid dysfunction affects and promotes the development of AD or the onset of AD leads to thyroid dysfunction remains controversial. In this study, we established an AD animal model and examined the serum and brain thyroid hormone and thyroid hormone receptors in the hippocampus, to further explore the relationship between the occurrence of AD and thyroid dysfunction.

## RESULTS

### AD model validation

The results of open field test showed that the residence time on the central area of the control group was significantly shorter than that of the experimental group (*P* < 0.05) (Table 1); The total distance of horizontal motion of the control group was significantly larger than the experimental group (*P* < 0.05) (Table 1); The recognition time of new objects in the experimental group was significantly longer than that in the control group (*P* < 0.05) (Table 1). However, these three results did not show significant differences between the control and normal mice.

**Table 1:** Results of behavioral experiments in mice (Mean ± Std., *n* = 6).

**Group**	**Retention time of in the central** **region of open field (sec)**		**Total distance of horizontal** **movement in open field (cm)**		**New object recognition time** **(sec)**	
	**F = 16.39, *P* = 0.005**		**F = 17.28, *P* < 0.001**		**F = 14.06, *P* < 0.001**	
Normal	120.4 ± 23.4	^#^*P* = 0.631	99.0 ± 5.9	^#^*P* = 0.12	65.6 ± 4.7	^#^*P* = 0.767
Control	109.3 ± 13.9		93.0 ± 4.2		63.8 ± 14.0	
OA	226.4 ± 62.0	^*^P < 0.001	78.2 ± 8.2	^**^P < 0.001	91.1 ± 8.9	^***^P < 0.001

^*^P, ^**^P and ^***^P: The *P* value of OA group was less than 0.001 compared with normal group and control group; ^#^*P*: Comparison between normal group and control group. Normal: Mice without stereotactic puncture; control: The mice were punctured stereotaxically and injected with 5 μL of normal saline; OA: Mice were subjected to stereotactic puncture and injection of 5 μL of 0.1 μM okadaic acid. (LSD test).

### Aβ and P-tau in the hippocampus region

**Figure 1:**
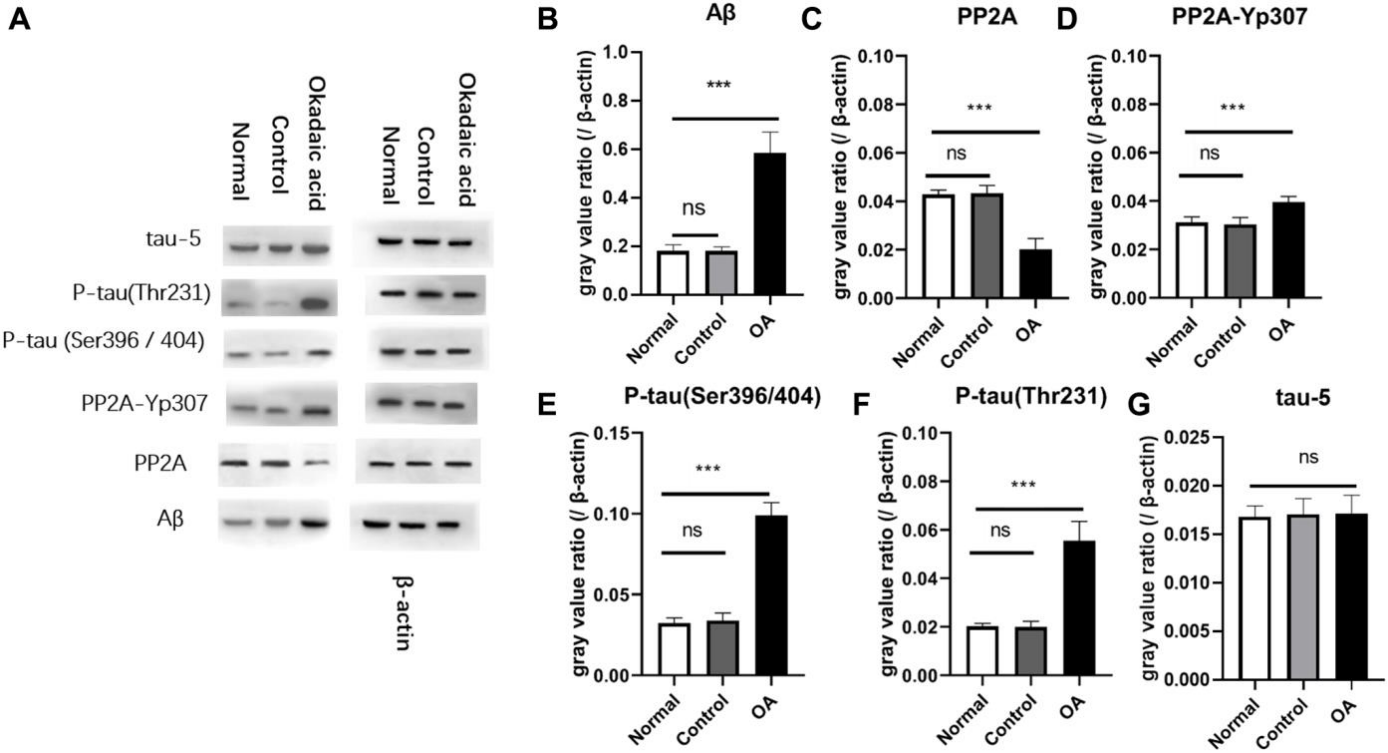
**Aβ and P-tau expression in mice.** Two weeks after stereotaxic injection, the expression of Aβ and P-tau in the hippocampus of the three groups of mice were detected by Western blot (**A**). The gray value analysis showed that there was no significant difference in the expression of Aβ between the normal group and the control group, and were significantly lower than the okadaic acid (OA) group (**B**) (ns: *P* = 0.985; ^***^P < 0.001) (*n* = 6, LSD test). The expression of PP2A of OA group was obviously lower than that of the normal group and the control group (**C**), but there is no difference between the normal and control group (ns: *P* = 0.792; ^***^P < 0.001). Compared with the normal group and the control group, the PP2A-Yp307 (**D**) (^***^P < 0.001), P-tau (Ser396/404) (**E**) (^***^P < 0.001) and P-tau (Thr 231) (**F**) (^***^P < 0.001) of OA group were significantly higher, but there was no significant difference between the normal group and the control group and the *P* values were 0.582, 0.936 and 0.600 respectively. The expression of tau-5 (**G**) was not significantly different among the 3 groups (ns, *P* = 0.724 and 0.799). Normal: Mice without stereotactic puncture; control: The mice were punctured stereotaxically and injected with 5 μL of normal saline; OA: Mice were subjected to stereotactic puncture and injection of 5 μL of 0.1 μM okadaic acid. (LSD test, *n* = 6).

Western blot assay and gray value analysis showed Aβ was significantly increased in the hippocampus of the mice after OA injection (Figure 1). In addition, to further confirm the expression of Aβ in the hippocampal region of mice, immunofluorescence detection of paraffin sections in the hippocampal region was supplemented (Supplementary Figure 1), and the results showed that the expression of Aβ in the hippocampal region was significantly increased in the experimental group (Supplementary Materials). The protein phosphotyrosine 307 phosphorylated PP2A (PP2Ac-yp307), P-tau (Ser396/404), P-tau (Thr231) in the control and normal group were significantly lower than those of OA group (*P* < 0.05), while protein phosphatase 2A (protein phosphatase 2A, PP2A) showed significant lower expression in OA group than that of the control and normal group control (*P* < 0.05). There was no difference of the expression of tau-5 among the three groups (*P* > 0.05). These differences were not found between the control and normal groups (*P* > 0.05) (Figure 1).

### Results of enzyme-linked immunosorbent assay (Elisa)

The results of Elisa showed that the levels of FT3, FT4, TSH, and TRH in brain tissue of OA group were higher than those of control group and normal group (*P* < 0.05), but there was no difference between control group and normal group (*P* > 0.05) (Table 2). The levels of FT4, TSH and TRH in serum of OA group were higher than those of control group and normal group (*P* < 0.05), but the levels of FT3 were not different from those of control group and normal group (*P* > 0.05), there was no difference in the levels of thyroid-related hormones between the control group and the normal group (*P* > 0.05) (Table 3).

**Table 2:** Thyroid hormone levels in brain tissue (Mean ± Std, *n* = 6).

**Group**	**FT3 (pg/mL)**	**FT4 (ng/mL)**	**TSH (μU/mL)**	**TRH (pmol/L)**
Normal	3.07 ± 0.77	0.96 ± 0.07	0.96 ± 0.24	4.61 ± 0.35
Control	3.24 ± 0.72	0.97 ± 0.04	1.05 ± 0.08	4.77 ± 0.33
OA	5.57 ± 2.10	1.29 ± 0.07	1.63 ± 0.26	5.72 ± 0.23
F, *P*	6.39, 0.010	52.83, <0.001	18.24, <0.001	25.25, <0.001
Intergroup comparison *P*	^*^P, ^#^0.836	^**^P, ^#^0.802	^***^P, ^#^0.475	^****^P, ^#^0.163

^*^P: the *P* values were 0.006 and 0.009, respectively, compared with the normal and control groups; ^**^P, ^***^P and ^****^P: The *P* value of OA group was less than 0.001 compared with normal group and control group; ^#^Comparison between the normal group and the control group. Normal: Mice without stereotactic puncture; control: The mice were punctured stereotaxically and injected with 5 μL of normal saline; OA: Mice were subjected to stereotactic puncture and injection of 5 μL of 0.1 μM okadaic acid. (LSD test).

**Table 3:** Levels of thyroid hormone in the serum (Mean ± Std, *n* = 6).

**Group**	**FT3 (pg/mL)**	**FT4 (ng/mL)**	**TSH (μU/mL)**	**TRH (pmol/L)**
Normal	3.19 ± 0.20	1.05 ± 0.14	1.13 ± 0.19	3.55 ± 0.12
Control	2.98 ± 0.30	0.95 ± 0.14	1.08 ± 0.08	3.52 ± 0.17
OA	3.11 ± 0.40	1.56 ± 0.23	1.66 ± 0.36	4.48 ± 0.21
F, *P*	0.698	21.13, <0.001	10.08, 0.001	61.14, <0.001
Intergroup comparison *P*	0.636, 0.258, ^#^0.500	^*^P, ^#^0.363	^**^P, ^#^0.744	^***^P, ^#^0.710

^*^P and ^***^P: The *P* value of OA group was less than 0.001 compared with normal group and control group; ^**^P: The *P* values were 0.002 and 0.001, respectively, compared with the normal and control groups; ^#^Comparison between the normal group and the control group. Normal: Mice without stereotactic puncture; control: The mice were punctured stereotaxically and injected with 5 μL of normal saline; OA: Mice were subjected to stereotactic puncture and injection of 5 μL of 0.1 μM okadaic acid. (LSD test).

### Expression of thyroid hormone receptors in hippocampus region of mice

**Figure 2:**
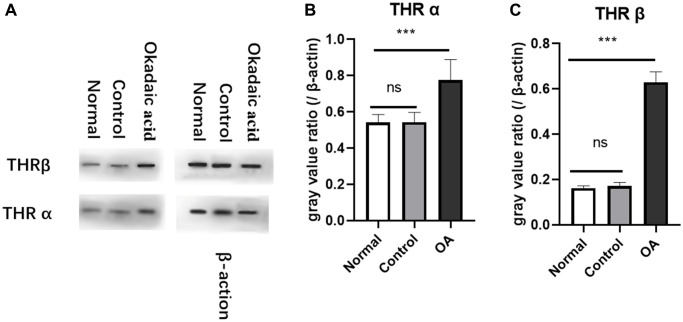
**Expression of thyroid receptors in hippocampus of mice.** Two weeks after stereotaxic injection, the expression of THRα and β in the hippocampus of the three groups of mice were detected by Western blot (**A**). The gray value analysis showed that the expression of THRα (**B**) and THRβ (**C**) protein were significantly higher than those of the normal and control group (^****^P < 0.001). However, there was no difference in the expression of THR between the control group and the normal group (*P* = 0.987 and 0.584, respectively). Normal: Mice without stereotactic puncture; control: The mice were punctured stereotaxically and injected with 5 μL of normal saline; OA: Mice were subjected to stereotactic puncture and injection of 5 μL of 0.1 μM okadaic acid. (LSD test, *n* = 6).

**Figure 3:**
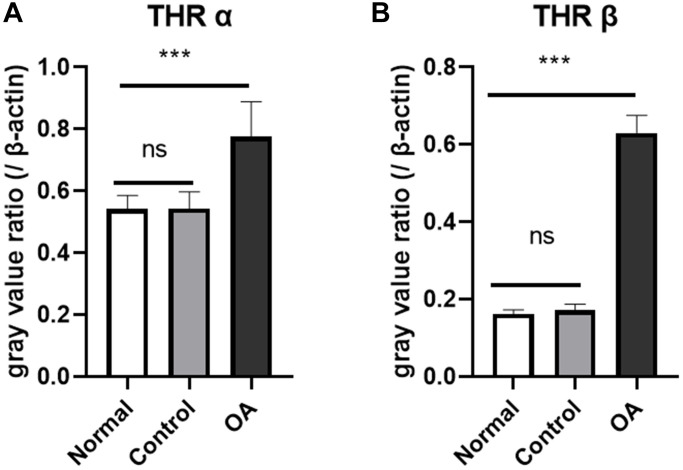
**Expression of thyroid hormone receptor gene in the hippocampus of mice.** The results of real-time quantitative PCR showed that the expression of thyroid receptor gene THRα (**A**) and THR β (**B**) was higher in the okadaic acid (OA) treated group than that in the control group at 2 weeks after injection (^****^P < 0.001), however, there was no significant difference in thyroid receptor gene expression between the control group and the normal group (*P* = 0.650 and 0.924, respectively). Normal: Mice without stereotactic puncture; control: The mice were punctured stereotaxically and injected with 5 μL of normal saline; OA: Mice were subjected to stereotactic puncture and injection of 5 μL of 0.1 μM okadaic acid. (LSD test, *n* = 6).

Western blot and gray value analysis showed that THRα and β in the hippocampus region of the experimental group were significantly higher than those of the control group and normal group, no difference was found between normal and control group (Figure 2). The quantitative polymerase chain reaction results also suggest an up-regulation of thyroid hormone receptor gene expression in the hippocampus region compared with the control group and normal group, but there was no significant difference between normal and control group (Figure 3).

## DISCUSSION

Okadaic acid (OA), a cytotoxin originally isolated from black sponges, is characterized by activation of GSK-3β, neuroinflammation and oxidative stress [18–20], and several *in vivo* and *in vitro* studies showed that OA induces tau phosphorylation, and can enhance the neurotoxicity of Aβ [21, 22]. Intraventricular administration of OA can cause memory impairment in laboratory animals in a water maze [23]; After low dose administration of 100 nM for 2 weeks, tau protein was induced at tau-S396 and tau-t231 sites in brain slices, but only weak phosphorylation occurred at tau-S199 sites, and tau-s396 and tau-t231 sites were induced by 100 nM at hyperphosphorylation [24]. In this study, one week after stereotactic injection of 100 nM OA into the hippocampus region, the behavioral abnormalities in open field and new object recognition test were consistent with those reported in the literature [22–24]. After two weeks, the expression of Aβ protein and the hyperphosphorylation of tau protein detected by western blot were consistent with the results reported in the literature [24]. Stereotactic puncture damage to hippocampal tissue may also cause potential behavioral changes in mice, therefore, in the design of the experiment, we compared the behavior of Aβ, P-tau, thyroid-related hormone and its receptor expression of the normal mice with that of the control mice, there were no significant differences in these data between normal and control mice, so it is possible that the damage to the hippocampus by the surgical puncture may be minimal or undetectable in the early stages [18].

Down regulation of protein phosphatase 2A (PP2A) activity plays a key role in the abnormal hyperphosphorylation of tau protein in AD. The inactivation of PP2A is caused by phosphorylation of its catalytic subunit (PP2Ac) tyrosine 307 (Yp307) [25, 26]. Our results showed that the activity of PP2A was down-regulated and the inactivated form of PP2Ac-yp307 was up-regulated in the mouse brain, which is consistent with the results of Liu et al. [25, 26]. These results further suggest that tau hyperphosphorylation and local amyloidosis in the brain via inhibition of protein phosphatase activity may be the mechanisms of OA induced AD in mice [21].

Changes in metabolic parameters are associated with an increased risk of dementia, in which thyroid function plays an important role in the pathology of AD. However, it is unclear whether thyroid dysfunction affects and promotes the initiation and/or progression of AD, or whether it is caused by AD. What is clear is that thyroid hormone plays an important role in normal brain learning and memory as well as neurogenesis, myelination and cell repair [2–7]. The increased levels of FT3, FT4 and TSH in the brain tissue of the experimental group may be because the repair response of the body after OA damage the brain tissue needs to increase the level of thyroid hormone to promote the repair of the damaged brain tissue. The body must secrete more TSH from the pituitary gland and more TRH from the hypothalamus to produce more TSH, which encourages the thyroid tissue to make more thyroid hormones [27, 28]. T3 had been shown to alter the splicing of APP genes and the secretion of this molecule in neuroblastoma cells, and to regulate the cellular and extracellular content of APP [29]. Evidence that T3 reduces β-amyloid precursor protein (APP) expression in neuroblastoma cells had also been reported, and that T3 negatively regulated APP gene expression [30]. In this study, the increased expression of Aβ amyloid protein in the experimental group required the increase of FT3 concentration in the brain to down-regulate the expression of APP gene, thus inhibiting the production of Aβ amyloid protein (which may be a repair response mechanism in mice) [28–30]. In addition, amyloidosis is mainly caused by the local administration of OA in the brain, while other organs or tissues with OA caused relatively minor damage. This may be the reason why peripheral serum FT3 showed no significant change compared with control mice, however, the changes of deiodinase in blood circulation and brain tissue need to be further examined.

The thyroid gland is responsible for the synthesis of the thyroid hormone (TH). TH production begins in the hypothalamus, where thyroid stimulating hormone releasing hormone (TRH) is released. TRH functions in the pituitary gland and stimulates the release of thyroid-stimulating hormone (TSH), whereas TSH regulates thyroid growth and hormone production. There are two active forms of TH, triiodothyronine (T3) and tetraiodothyronine or thyroxine (T4) [31]. T4 is a half-life longer than T3 and is considered to be the main form of TH in the cycle. From the total TH production, 93% was T4 and 7% was T3 [31]. T4 binds to protein in serum or to free T4 in free form (FT4). When T4 is in the tissue, it is converted to T3 by deiodinase, which is thought to be a metabolically active form of TH, or to reverse T3 (rT3), an inactivated form of T3 [32]. Finally, T3 binds to its nuclear TH receptors (TRs), before the target genes are repressively inhibited or activated [33]. Our results suggest that the increased expression of THRα and β and their genes in the brain tissue of the mice in the experimental group may also be the mechanism of the repair response in mice at the early stage of the AD model, but further evidence is needed, which is consistent with the results reported previously [34], but contrary to the results in another report [35], the most likely reason is that the hypothalamic-pituitary-thyroid axis modulates thyroid function by a negative feedback mechanism [36, 37]. When FT3 or FT4 is elevated, the negative feedback inhibits the concentrations of TSH and TRH and keeps the FT3 and FT4 of thyroid secretion in the normal range. FT3, FT4, and TSH in circulation are at normal levels in the middle and late stages of AD, but FT3 and FT4 in brain tissue are at low levels, while long term local low level FT3 may induce down regulation of local TRs protein and its gene expression, but further experiments are needed to confirm it.

Through this experiment, we hypothesized that after OA damaged the hippocampus of mice, the disturbance of brain and blood circulation in mice and the up-regulation of thyroid hormone receptor protein and gene expression may be the mechanism of early repair reaction in AD patients. The studies showed that peripheral and central administration of T3 could improve the histological changes, memory, and dentate electrical activity in a rat model of Alzheimer’s disease [30]. The shortcoming of this study is that deiodinase [31] activity in peripheral tissues was not detected, but the change of local deiodinase activity may also be another reason for the increase of FT3 in brain. This study provides some basic theoretical basis for the study on delaying the progression of AD mouse model by increasing the concentration of FT3 in brain, because similar experiments in rat models have been carried out with encouraging results [30].

## CONCLUSION

One week after a small dose of okadaic acid was stereotaxically injected into the hippocampus, the mice showed behavioral changes such as longer stays in the center of the open field, shorter horizontal movement distances, and increased time spent identifying new objects, which suggested that okadaic acid induced damage in the hippocampus of mice. The results of Aβ and P-tau detection 2 weeks after injection showed that okadaic acid mainly caused the high expression of Aβ and P-tau in the hippocampus of mice. Two weeks after injection of okadaic acid, there were significant differences in thyroid-related hormones and their receptors and their genes in brain tissue and serum between the mice and the control group (except serum FT4). There was no significant difference between the normal group and the control group. Therefore, we believe that the effect of surgical manipulation (stereotactic injection) injury on the hippocampal region in mice is minimal. A mouse model of AD could be successfully established by stereoscopic injection of a small dose of okadaic acid into the hippocampus; in the early days of the AD model, abnormal expression of thyroid-related hormones and receptors and genes in brain tissue and serum were not associated with surgical trauma (data not indicated).

## MATERIALS AND METHODS

### Experimental materials

Okadaic acid (Sigma-Aldrich Biotech Co., Ltd.), western bolt required antibody, reagent (Nanjing Jinsirui Biotechnology Co., Ltd.), thyroid hormone receptor (THR α, β) PCR kit (Yeasen Biotech Co., Ltd.), mice FT3, FT4, TSH Elisa Reagents (Beijing Labgic Technology Co., Ltd.), Fluorescence microscope (Nova View, INOVA Diagnostics, Inc.), SDS-PAGE electrophoresis and transmembrane system (Jinan Biobase Biotech), Multiskan FC enzyme marker (Thermo Scientific, Co., Ltd.), Open field experiment video analysis system (XR-XZ301, Shanghai Xinruan Information Technology Co., Ltd., Shanghai, China).

### Experimental animals

BALB/C mice purchased from Zunyi Medical University Laboratory Animal Center, 12 weeks old, 18 male mice, 25–30 g in weight, were raised at room temperature in Specific Pathogen Free environment (SPF), 12 hours photoperiod night cycle. All animal experiments are approved by the Animal Ethics Committee of Zunyi Medical University.

### Methods

#### Establishment of the AD model

The mice were randomly divided into normal group, control group and experimental group. After weighing, the mice in the control and experimental groups were anesthetized by intraperitoneal injection of 2% sodium pentobarbital (45 mg/kg). The incisors of the anesthetized mice were fixed to the maxillary fixator of the brain locator, and then one ear stick was pushed into the animal’s external auditory canal, placing the animal’s head in the middle of the two slideways, and the other ear stick was pushed into the other ear canal, after making sure that the scales of the two ear bars are the same, tighten the fixing screws on the ear bars, and then press the nose ring on the tooth fixer down and twist tightly; After the hair of the head was cut off and the skin of the head was disinfected with 2% iodine and 75% alcohol, a 3 cm skin incision was made along the sagittal suture to separate the subcutaneous tissue, the fascia and muscles on the surface of the skull were cleaned with hydrogen peroxide (H_2_O_2_), and the periosteum was removed, exposure of anterior fontanel, herringbone and sagittal suture; The metal locating needle was moved down to the top of sagittal suture, then moved the locating needle back and forth to position the locating needle to the anterior fontanel; A mark was made at the intersection of 2 mm posterior to the anterior fontanel and 2.5 mm adjacent to the sagittal suture (at the level of the hippocampus region) with the locating needle, and a small round hole about 1 mm in diameter was drilled on the skull with a drill needle; The control group was injected with 0.9% sodium chloride solution 5 μL, the experimental group was injected with 0.1 μM OA 5 μL, the injection speed was 1.0 μL/min, the needle was placed 5 minutes after injection, then the skin of mice was sutured and sterilized according to the above method, and the mice were kept for 2 weeks.

#### Open field experiment

The open field is usually divided into four corners, four sides, and a central area (a square with a center point at the bottom of the box and a quarter of the bottom area) (Supplementary Video 1). One week after injection, experiments were performed in a quiet environment, and mice were placed at the bottom surface center of the inner bin, along with phase capture and timing for 3–5 min. After the end of each experiment, the information left by the mice including urine, smell, etc. should be cleaned, in order to prevent these from potentially affecting the results of the experiment. The residence time (second) at the center of the open field and the total horizontal distance (cm) of the mice in the open field were recorded.

#### New object recognition

Before the new object recognition experiment, the experimental animals were placed in the new object recognition experimental system for 24 hours, then the new objects were placed in the experimental environment, and the mice were placed in the experimental system, a video recording device was activated to record the time the mice were exposed to the new object (each trial lasted 10 minutes) (Supplementary Video 2).

#### Detection of FT3, FT4, TSH and TRH

Two weeks after injection, the blood of mice was collected through the orbital vein collection method, then the mice were sacrificed by the cervical dislocation method, the brain tissue of the hippocampus area of the mice was separated, fixed and preserved for use, and then the remaining brain tissue was made into homogenate, the concentration of FT3, FT4, TSH and TRH in serum and brain tissue homogenate were determined by Elisa.

#### Protein isolation and western blot analysis

The operation of protein isolation and western blot analysis were based on previous literature reports [16, 17]. Briefly, the whole process was performed on ice or at 4°C, the cerebral cortex of hippocampal region (about 0.5 g) was taken, then 1–2 mL of RIPA and phosphate protease inhibitor were added, homogenate at 4°C for 10 minutes; followed by ultrasonic cracking for 20 minutes, centrifuging at 10000 rpm and 4°C for 10 minutes, discarding the supernatant and the protein concentration was determined by BCA method, the sample buffer was boiled at 99°C for 10 minutes, and then stored at −80°C for the following experiment. For western blot analysis, 60 μg of protein from each sample was resolved by electrophoresis on a 10% polyacrylamide gel. The proteins on the gel were transferred into an SDS-PAGE electrophoresis and transmembrane system. After incubating with 5% bovine serum albumin (BSA) for 1 h, the membrane was probed with the primary antibodies (1:500), blots were developed using metal enhanced DAB substrate kits.

#### Real-time quantitative polymerase chain reaction (Q-PCR)

The operation of Q-PCR is briefly described according to reference [18] and was briefly described as follows: The whole experiment was carried out on ice or at about 4°C, the RNA of the cortex (about 0.5 g) was extracted after adding Tizol, and then the extracted RNA was transcribed into cDNA according to the references [19]; Forward and reverse primer sequences were designed in accordance with published sequences available in references [19, 20]. The real-time quantitative polymerase chain reaction was performed in SsoAdvanced SYBR^®^ Green Supermix (Bio-Rad, France) instrument.

### Statistical methods and image analysis

All measurement data are indicated by mean plus minus standard deviation (Mean ± Std.); Comparisons between means were made using SPSS 18.0 One-way ANOVA and LSD-t test, setting the test level to *P* < 0.05, and the gray value of the images was quantified using Image J software.

## Supplementary Materials

Supplementary Methods

Supplementary Figure 1

Supplementary Video 1

Supplementary Video 2
