# Supplementary material for: The disturbance of thyroid-associated hormone and its receptors in brain and blood circulation existed in the early stage of mouse model of Alzheimer’s disease
Source: Aging (Albany NY). 2023 Mar 7;15(5):1591–602. doi: 10.18632/aging.204570 (PMC10042683; doi:10.18632/aging.204570)
Supplement: Supplementary Methods [file aging-15-204570-s001.pdf]

SUPPLEMENTARY METHODS

Immunofluorescence staining was carried out according to the method reported in reference [24], which was briefly described as follows: the tissue of the hippocampus region of mice was removed from the refrigerator at −80°C and immediately soaked in 4% paraformaldehyde solution for 48 h, then embedded in paraffin wax and prepared into sections of 5 μm thickness. After the sections were dewaxed and dehydrated, the sections were placed in citrate (pH 6.0) antigen repair solution for hot repair for 15 min. After the sections were naturally cooled to room temperature, the sections were incubated with 3% H_2O_2 for 15 min. Then the sections were closed with goat serum and added primary antibody Aβ (1:500) (polyclonal, Invitrogen), 4°C overnight and 45 min after rewarming, FITC-labeled secondary antibodies (1: 500) (Polyclonal, Bioworld) were slowly shaken and incubated on a side shaking bed at room temperature for 1 hour, washed with PBS 3 times, 5 minutes each time, and stained with DAPI (10 minutes at room temperature, washed with PBS 3 times, 5 minutes each time). Finally, the tablets were sealed with anti-fluorescence quench agent.
