# Supplementary material for: The disturbance of thyroid-associated hormone and its receptors in brain and blood circulation existed in the early stage of mouse model of Alzheimer’s disease
Source: Aging (Albany NY). 2023 Mar 7;15(5):1591–602. doi: 10.18632/aging.204570 (PMC10042683; doi:10.18632/aging.204570)
Supplement: Supplementary Figure 1 [file aging-15-204570-s002.pdf]

SUPPLEMENTARY FIGURE

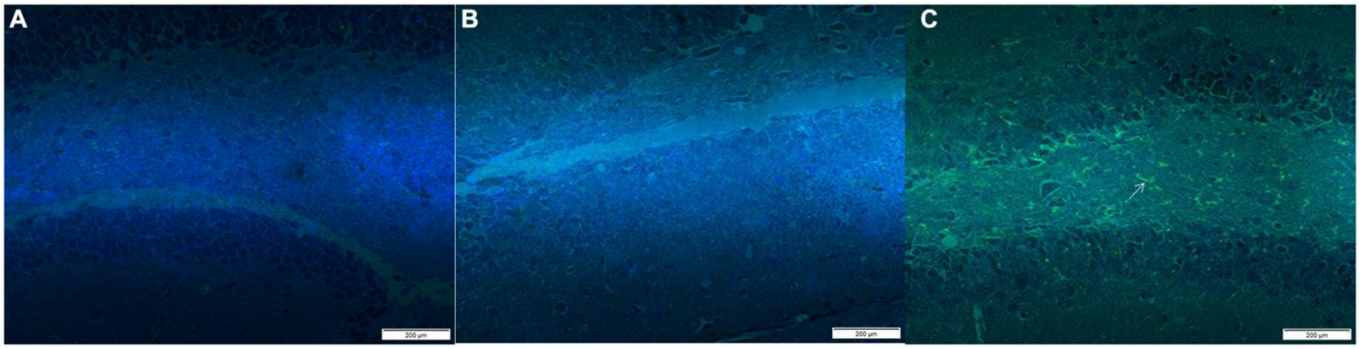

Supplementary Figure 1. Results of immunofluorescent staining of Aβ in hippocampus of mice. After the injection of okadaic acid (0.1 μM, 5 μL) into the hippocampus for 2 weeks, the expression of Aβ in the hippocampus of mice showed little irregular patchy green fluorescence in the interstitium of the normal group (**A**) and the control group (**B**), while the irregular patchy green fluorescence was abundant and strong in the interstitium of the OA treatment group (**C**). Normal: mice without directional puncture; Control group: Mice were injected with 5 μL normal saline through stereotactic puncture. (x 20).
